# Supplementary material for: Time‑resolved multi-omic analysis of paclitaxel exposure in human iPSC‑derived sensory neurons unveils mechanisms of chemotherapy‑induced peripheral neuropathy
Source: Cell Death Dis. 2026 Feb 10;17(1):211. doi: 10.1038/s41419-026-08445-2 (PMC12921266; doi:10.1038/s41419-026-08445-2)
Supplement: Supplementary file 15 — Dataset 1. [file 41419_2026_8445_MOESM15_ESM.zip › CDD_Open_Data/04_RNA_Seq/Timeline_panel_plots_etc_2023-01-23_1609.zip!/Panel_Plots_etc/Evidence_Plots/post5d/evidence_plot_standard contrasts_msigdb_reactome_5d_PTX_vs_DMSO_ID6_M16227_REACTOME_CHOLESTEROL_BIOSYNTHESIS.pdf]

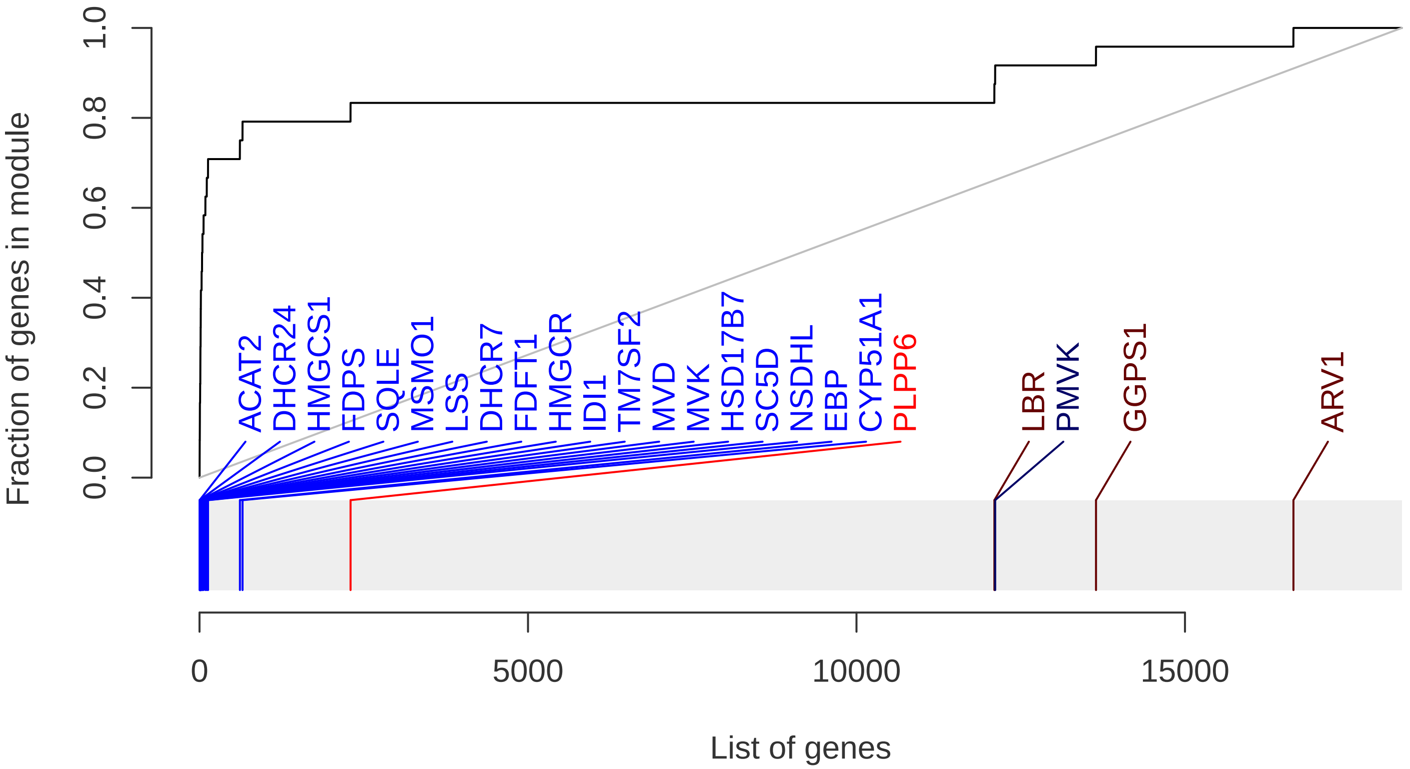
Fraction of genes in module
0.0
0.2
0.4
0.6
0.8
1.0
ACAT2
DHCR24
HMGCS1
FDPS
SQLE
MSMO1
LSS
DHCR7
FDFT1
HMGCR
IDI1
TM7SF2
MVD
MVK
HSD17B7
SC5D
NSDHL
EBP
CYP51A1
PLPP6
LBR
PMVK
GGPS1
ARV1
0
5000
10000
15000
List of genes
